# Effects of Pain Relief on Arterial Blood O_2_ Saturation

**DOI:** 10.5812/traumamon.14034

**Published:** 2014-01-25

**Authors:** Hossein Alimohammadi, Alireza Baratloo, Ali Abdalvand, Alaleh Rouhipour, Saeed Safari

**Affiliations:** 1 Department of Emergency Medicine, Imam Hosein Hospital, Shahid Beheshti University of Medical Sciences, Tehran, IR Iran; 2 Department of Emergency Medicine, Shohadaye Tajrish Hospital, Shahid Beheshti University of Medical Sciences, Tehran, IR Iran; 3 Department of Family Medicine, University of Alberta, Edmonton, Canada; 4 Department of Pediatrics, Valeeasr Hospital, Ghazvin University of Medical Sciences, Abyek, IR Iran

**Keywords:** Wounds and Injuries, Pain, Pulse Oximetry, Respiratory Rate, Fentanyl

## Abstract

**Background:**

Pain management with the use of sedatives and analgesics has several advantages and few complications or side effects.

**Objectives:**

In this study, we planned to evaluate the effects of pain control on oxygen saturation independent of other factors, such previous cardio-pulmonary conditions or respiratory rate.

**Patients and Methods:**

Sixty-seven adult patients with direct trauma to extremities, who were referred to Imam Hossein Educational Hospital emergency room were enrolled in this study. Exclusion criteria were trauma to parts of the body other than extremities, and comorbidity with cardiovascular, pulmonary, or other disorders. Pain was evaluated using a numerical rating scale and scored between 0-10. Patients’ respiratory rates (RR) were recorded by a physician and blood oxygen saturations were measured using a pulse oximeter. Then, fentanyl 1 μg/kg was administered under direct supervision of a physician. After five minutes, pain score, oxygen saturation, and RR were measured in the above-mentioned order.

**Results:**

The data from 67 patients with a average age of 30 years were collected: 77% were male and 23% were female. The average pain score of these patients was 7.3 at the time of admission, which significantly decreased to 3.8 after fentanyl administration (P < 0.001). Upon arrival in emergency department the mean oxygen saturation and RR were 97.1% and 21.5/minute, respectively. After pain control, mean oxygen saturation and RR were 94.9% and 19.2 /minute, respectively, showing a significant decrease only for RR in comparison with that at the time of admission (P < 0.001). Regression analysis of pain score and O_2_ saturation differentiation showed no significant relation between these variables. There were no side effects or complications of fentanyl observed in these patients.

**Conclusions:**

The results of our study revealed no independent causative relationship between pain control and oxygen saturation.

## 1. Background

Fear of respiratory depression often causes pain to be left undertreated and patients experience unnecessary pain (1). In spite of increased use of different analgesics, opioids continue to be the mainstay for pain management in a wide variety of clinical situations around the world. Meanwhile, there are still concerns about their potential adverse effects on ventilation (2). Integrating opioid risk and benefit into a single function may give a useful single measurement method for positive and negative effects of opioids (3). Although rare, potential opioid-induced respiratory depression is a major limiting factor for provision of effective analgesia. Therefore, it is of paramount importance to achieve sufficient analgesia with minimal side effects; although this usually involves a combination of therapeutic approaches, opioids remain the backbone of pain therapy (1, 4). Unintended increase of sedation and respiratory depression are two of the most serious opioid-related adverse events; the most commonly used term “respiratory depression” describes only a part of that risk. Opioid-induced ventilatory impairment (OIVI) is a more complete term encompassing opioid-induced central respiratory depression (decreased respiratory drive), decreased level of consciousness (sedation), and upper airway obstruction, all of which, alone or in combination, may result in decreased alveolar ventilation and increased arterial carbon dioxide levels (2, 5). As the complexity of analgesic therapy increases, priorities of care must be established to balance aggressive pain management with measures to prevent or minimize adverse events and ensure safe care. Multiple factors, including opioid dosage, route of administration, duration of therapy, patient-specific factors and desired goals of therapy, can influence occurrence of these adverse events (5).

## 2. Objectives

In this study, we evaluated the effects of pain control on oxygen saturation independent of other factors such as cardio-pulmonary conditions or respiratory rate.

## 3. Materials and Methods

In this study, 67 adult patients with isolated and direct trauma to extremities referred to the emergency department of our hospital were studied. The study protocol was accepted by the ethics committee of Shahid Beheshti University of Medical Sciences and the principles of the Declaration of Helsinki were observed throughout the study. Patients with trauma to any part of the body other than the extremities, and those suffering from cardiovascular, pulmonary, or other co-morbid disorders were excluded from the study. A data collection sheet was designed to gather demographic data as well as pain score, respiratory rate (RR), and blood oxygen saturation which were checked by a physician before and after analgesic administration. Pain was evaluated using a numerical rating scale and scored 0-10. Patient’s respiratory rate was recorded by a physician and blood oxygen saturation was measured using a finger pulse-oximeter (Nonin 9550). Afterwards, fentanyl 1 μg/kg was administered under direct supervision and observation of a physician. After five minutes (when fentanyl effect reached its peak level), pain score, oxygen saturation, and RR were measured in the aforementioned order. Any decrease in the level of conciouseness, appearance of side effects, or complications were deemed reasons to stop the assessment. Since we only selected patients with direct trauma to the extremities, supplemental oxygen was not administered. Data analysis was performed using SPSS 18.5 (SPSS, Chicago, Illinois). Paired t test was used to compare continuous variables (O_2_ saturation and pain scores) during, before, and following fentanyl administration. A stepwise linear regression model was developed to identify the association between O_2_ saturation and pain severity scores. P < 0.05 was considered statistically significant.

## 4. Results

The data from 67 patients with an average age of 30 years were collected (77% male). The mean pain score of these patients at the time of admission was 7.3 (min:4, max:10), which significantly decreased to 3.8 (min:0, max:10) after fentanyl administration (P < 0.001). The mean respiratory rate before and after pain control were 21.5/minute (min: 14, max: 30) and 19.2 /minute (min: 13, max: 28), respectively, showing a significant decrease in comparison at the time of admission (P < 0.001) (Table 1). 

**Table 1.** Respiratory Rate and Pain Score Before and After Fentanyl Administration

	Before Fentanyl Administration	After Fentanyl Administration	P value
**Respiratory rate/min**	21.5	19.2	< 0.001
**Pain score**	7.3	3.8	< 0.001

The mean oxygen saturation was 97.1% (min: 89, max: 100) upon arrival in the emergency department. After pain control, mean oxygen saturation was 94.9% (min: 82, max: 100). In spite of significant decrease in post-analgesic RR, arterial blood saturation did not show a significant decrease (P > 0.05). Regression analysis of pain score and oxygen saturation differentiation showed no significant correlation between these variables (Table 2). No side effects or complications of fentanyl were observed in the patients.

**Table 2.** Association of Hemoglobin, O_2_ Saturation and Pain Score

Pain Score	Beta	95% Confidence Interval	P value^a^
**Before fentanyl administration**	0.21	[-0.4 - 0.8]	0.5
**After fentanyl administration**	-0.20	[-0.69 - 0.28]	0.4

^a^ Based on stepwise linear regression

## 5. Discussion

In 2011, the American Society for Pain Management claimed that despite the frequency of opioid- induced sedation, there are no universally accepted guidelines to direct effective and safe assessment and monitoring practices for patients receiving opioid analgesia. Moreover, there is a paucity of information and no consensus regarding the benefits of technology-supported monitoring, such as pulse oximetry (measuring oxygen saturation) and capnography (measuring end-tidal carbon dioxide) in hospitalized patients receiving opioid for pain therapy (5). Macintyre and colleagues in 2011 stated that the goal of acute pain management requires that opioid administration should be titrated. However, methods aiming appropriate pain scores without the need for OIVI monitoring may lead to an increase in adverse incidents. Therefore, all patients should be monitored for OIVI (at the very least using sedation scores as the “6th vital sign”), in order to note complications as soon as they occur (2). While there is no universally accepted standard protocol for monitoring opioid administration, pulse oximetry is usually enough. Oxygen saturation of the blood (SaO_2_) is determined mainly by arterial partial pressure of oxygen (PaO_2_). The relationship between these two variables is the known oxygen dissociation curve, which was determined experimentally by in vitro titration of the blood while increasing oxygen partial pressures. At low oxygen pressures, there is relatively little increase in SaO_2_ for a given change in PaO_2_. For PaO_2_ above 20 mmHg, the rate of SaO_2_ change increases markedly and then drops beyond PaO_2_ of 60 mmHg. PaO_2_ is the most important (but not the only) determinant of SaO_2_. Boom and colleagues in a recent study mentioned that fentanyl causes marked respiratory depression and analgesia (3). The pharmacokinetic and pharmacodynamic models sufficiently described the data. Usefulness efficiency on the basis of fentanyl's experimental impressions on respiration and pain relief , were assessed. These results were helpful in numerous comparisons between experimental drugs. Complementary studies are necessary to assess whether or not this risk-benefit analysis is valuable in clinical settings (3). In our study with 1 μg/kg fentanyl , none of the side effects or complications of fentanyl were observed in the patients; although we did not measure its serum concentration. Pain control was achieved in all patients. Smith and colleagues in 2010 explained that pharmacodynamic interactions could aggravate the respiratory depressive effects of opioids and thus compromise their safety. Patients with impaired renal or hepatic function or multi-drug use may have trouble in clearance or metabolization of opioids leading to increased risk of adverse events. Patients with cerebrovascular, cardiovascular, or respiratory diseases (including smokers of more than two packs per day) may be predisposed to bradycardia, hypotension, and respiratory depression with any opioid; some particular opioids may pose additional risks. Patients with brain injury, dementia, psychiatric illness , or cerebrovascular disease are more predisposed to opioid side effects on the central nervous system (CNS), which include cognitive impairment, sedation, and euphoria. Suitable opioid choice may diminish these effects. This review brings about an overview of opioid consumption in medically problematic patients and recommendations on how to establish appropriate analgesia while keeping above adverse reactions and drug interactions in clinical conditions (6). Pergolizzi and colleagues in 2008 also mentioned that respiratory depression was a serious threat in patients receiving concomitant CNS drugs associated with hypoventilation, or with underlying pulmonary conditions requiring pain management. They also stated that different opioids did not show equal influences on ventilation, and named buprenorphine as the only opioid demonstrating a ceiling for respiratory depression. Therefore, different features of opioids regarding respiratory effects should be considered when treating patients at risk for respiratory problems (7). Hence, we excluded patients with cardiovascular, pulmonary, or other co-morbid disorders to decrease possible errors. In a review article conducted by Pattinson in 2008, it was advocated that pain stimulates respiration (8, 9). Substance P and NK-1 receptors mediate nociception and respiration, and therefore, such a close correlation between breathing and pain is not surprising. Indeed, several brainstem sites express opioid receptors such as the ventral medulla and parabrachial complex areas chemoreceptive and nociceptive sites (10). It is assumed that pain increases tonic input to the respiratory centers, rather than enhancing chemoreflex sensitivity (10). The clinical implication of this common pathway between pain and respiration would be situations where patients receiving sub-therapeutic doses of opioids exhibit spontaneous breathing. In these cases, the use of subsequent neuraxial block can cause severe respiratory depression (1). It seems that pain relief after opioid administration does not have statistically significant effects on arterial blood saturation changes. Moreover, our present work was a pilot study to assess the effects of opioid analgesia on ventilation and the pattern of possible complication of this pain management method.
